# Deep phenotyping of myalgic encephalomyelitis/chronic fatigue syndrome in Japanese population

**DOI:** 10.1038/s41598-020-77105-y

**Published:** 2020-11-16

**Authors:** Toshimori Kitami, Sanae Fukuda, Tamotsu Kato, Kouzi Yamaguti, Yasuhito Nakatomi, Emi Yamano, Yosky Kataoka, Kei Mizuno, Yuuri Tsuboi, Yasushi Kogo, Harukazu Suzuki, Masayoshi Itoh, Masaki Suimye Morioka, Hideya Kawaji, Haruhiko Koseki, Jun Kikuchi, Yoshihide Hayashizaki, Hiroshi Ohno, Hirohiko Kuratsune, Yasuyoshi Watanabe

**Affiliations:** 1 RIKEN Center for Integrative Medical Sciences, Kanagawa, Japan; 2 Osaka City University Graduate School of Medicine, Osaka, Japan; 3 Kansai University of Welfare Sciences, Osaka, Japan; 4 RIKEN Center for Biosystems Dynamics Research, Hyogo, Japan; 5 Nakatomi Fatigue Care Clinic, Osaka, Japan; 6 RIKEN Compass to Healthy Life Research Complex Program, Hyogo, Japan; 7 RIKEN Baton Zone Project, RIKEN-JEOL Collaboration Center, Hyogo, Japan; 8 RIKEN Center for Sustainable Resource Sciences, Kanagawa, Japan; 9 RIKEN Preventive Medicine and Diagnosis Innovation Program, Saitama, Japan; 10 Graduate School of Bioagricultural Sciences, Nagoya University, Aichi, Japan; 11 Graduate School of Medical and Life Sciences, Yokohama City University, Kanagawa, Japan

**Keywords:** Fatigue, Diagnostic markers

## Abstract

Myalgic encephalomyelitis/chronic fatigue syndrome (ME/CFS) is a complex and debilitating disease with no molecular diagnostics and no treatment options. To identify potential markers of this illness, we profiled 48 patients and 52 controls for standard laboratory tests, plasma metabolomics, blood immuno-phenotyping and transcriptomics, and fecal microbiome analysis. Here, we identified a set of 26 potential molecular markers that distinguished ME/CFS patients from healthy controls. Monocyte number, microbiome abundance, and lipoprotein profiles appeared to be the most informative markers. When we correlated these molecular changes to sleep and cognitive measurements of fatigue, we found that lipoprotein and microbiome profiles most closely correlated with sleep disruption while a different set of markers correlated with a cognitive parameter. Sleep, lipoprotein, and microbiome changes occur early during the course of illness suggesting that these markers can be examined in a larger cohort for potential biomarker application. Our study points to a cluster of sleep-related molecular changes as a prominent feature of ME/CFS in our Japanese cohort.

## Introduction

Myalgic encephalomyelitis/chronic fatigue syndrome (ME/CFS) is a complex and debilitating disease with a spectrum of symptoms including unexplained fatigue, post-exertional malaise, impaired memory, pain, gastrointestinal and immune dysfunction, and sleep disturbance^1,2^. About 0.2 to 2.6% of the population, 75% being female, are estimated to be affected by ME/CFS^3,4^ with no treatment option, resulting in depression, absence from work, and social isolation. ME/CFS is currently diagnosed based on symptoms and there are currently no accepted molecular diagnostic tools^5^. This poses challenges to medical practitioners who often rely on molecular diagnostic tools and physical signs of illness for making diagnostic decisions.

In order to identify potential diagnostic markers for ME/CFS, researchers have relied on omics technologies to systematically profile molecular changes associated with ME/CFS. One of the first omics technologies to be applied was microarray, which measures the gene expression changes across thousands of genes in the genome. This technology led to the identification of gene expression markers for ME/CFS, particularly in peripheral blood mononuclear cells (PBMCs)^6,7^. However, a validation study in a different cohort failed to robustly separate patients from controls^8^. Additionally, a study in twin pairs has shown that no gene expression difference could be detected between ME/CFS patients and controls when controls were genetically matched^9^, suggesting that gene expression changes do not strongly reflect the disease state of ME/CFS.

The use of omics technologies in ME/CFS studies have recently shifted to metabolomics and microbiome analysis. Metabolomics studies using gas chromatography–, liquid chromatography–, or capillary electrode–mass spectrometry (GC–MS, LC–MS, or CE–MS) in plasma samples have identified sets of metabolites that can robustly distinguish ME/CFS patients from healthy controls^10–14^. However, more studies are needed to identify common sets of metabolites that are altered in ME/CFS patients as the number of studies are still few. In comparison to metabolomics study, a larger number of fecal microbiome analysis have been conducted for ME/CFS, which is summarized in a recent review^15^. Among the key differences detected, a decrease in *Faecalibacterium* appears to be shared across several studies of ME/CFS. In addition to these technologies, global cytokine profiling^16–19^ and immunophenotyping^20,21^ have been performed in plasma and cerebrospinal fluid samples to identify immune biomarkers of ME/CFS. More recently, a proteomics survey of extracellular vesicles (EVs) was performed to identify EV-specific protein biomarker of ME/CFS^22^. All of these technologies survey biological samples from the periphery, which are readily accessible to medical practitioners.

In parallel to the advancement in omics technologies targeting the periphery, advances in imaging technologies have revealed key insights into molecular changes that occur in the brain of ME/CFS patients^23–29^. These studies have uncovered neuroinflammation^30^ related to the microglial activation by cytokines, neurotransmitter abnormalities^31,32^, costly and less efficient performance of frontal cortex^33^, as well as ‘brain fog’^34^. These imaging technologies require trained specialists to perform the study and omics technologies still represent a more accessible platform for molecular diagnostics in most laboratories.

Despite the accumulation of omics data in ME/CFS, key challenges remain in integrating and interpreting these data. First, the performance of omics platforms cannot be compared to each other as the studies are conducted in different sets of cohorts. Second, one cannot relate changes in molecular profile at one omics level, such as metabolite, to another omics level such as microbiome, when patients differ between studies. Therefore, in order to address these challenges, we set out to perform a deep phenotyping of ME/CFS using five molecular profiling platforms, accompanied by questionnaire and quantitative measures of fatigue. From our study, we identified 26 potential markers that distinguished ME/CFS patients from healthy controls. We found that markers from immunophenotyping, microbiome analysis, and lipoprotein profiling performed best in distinguishing patients from controls. When combined, these markers did not completely separate patients from controls suggesting limitations of our profiling technologies. We also uncovered strong correlation between sleep disruption, lipoprotein profiles, and microbiome abundance suggesting that these markers form one of the core networks of ME/CFS. These changes were evident during the early course of illness suggesting that these markers may be studied in a larger cohort for biomarker application. Our study points to a cluster of sleep-related molecular changes as a prominent feature of ME/CFS in our Japanese cohort. 

## Results

### Overview of the study

**Figure 1:**
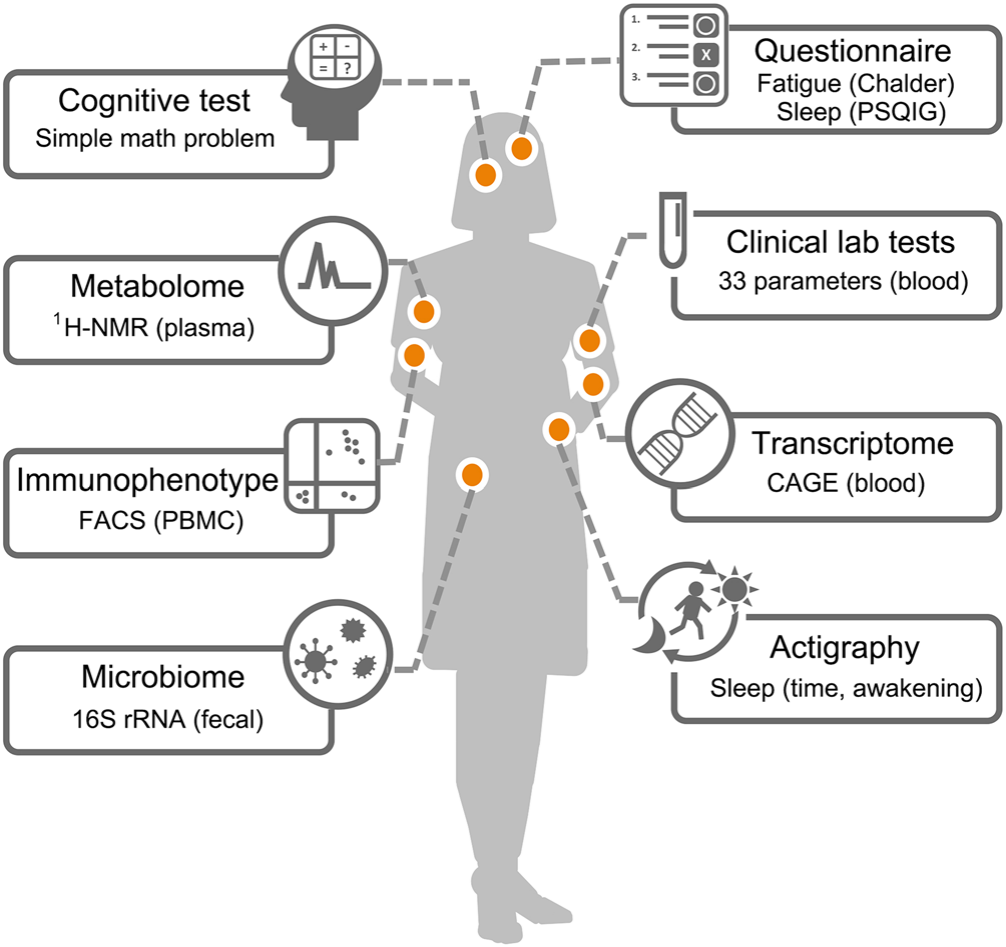
Deep phenotyping of myalgic encephalomyelitis/chronic fatigue syndrome (ME/CFS). Schematics of the datasets collected from myalgic encephalomyelitis/chronic fatigue syndrome (ME/CFS) patients and healthy controls.

The goal of our study was twofold: (1) identify potential markers of myalgic encephalomyelitis/chronic fatigue syndrome (ME/CFS) across multiple omics platforms; and (2) uncover relationships between these markers for insights into the syndrome. We recruited 48 ME/CFS patients and 52 healthy controls (Table S1) matched for age, gender, and BMI. ME/CFS patients were diagnosed based on the 1994 Center for Disease Control clinical criteria (Fukuda criteria)^1^ and the International Consensus Criteria^2^. These cohorts underwent questionnaires, activity measurements, simple cognitive tests, as well as multi-omics profiling of blood and fecal samples (Fig. 1). To assess the severity of fatigue, we used Chalder fatigue scale^35^ and quality of sleep using Pittsburgh Sleep Quality Index Global (PSQIG) score^36^. To quantitatively measure sleep, we also performed 1-week actigraphy measurements^37^. For cognitive assessment, we administered simple mathematical problems. For molecular profiling, blood samples were taken in the morning after overnight fast and assessed for standard clinical laboratory tests, ^1^H-NMR metabolomics of plasma, FACS-based immunophenotyping of peripheral blood mononuclear cell (PBMC), and transcriptome analysis of whole blood. Fecal samples were collected at home and were sent for analysis of microbiota composition using 16S rRNA sequencing. Given the technical difficulties in several of the omics measurements as well as difficulties in obtaining enough patient materials, some of the measurements could not be completed (Fig. S1). For each platform, we used all available data to identify markers that distinguished ME/CFS patients from healthy controls. To compare markers across platforms, we restricted the analysis to individuals with complete data. In total, we evaluated 33 standard clinical laboratory tests (Table S1), 20 types of immune cell (Fig. S2), 31 metabolite profiles (Table S2), eight lipoprotein fractions (Table S3), expression levels of 820 gene sets, and relative abundance of 20 different bacterial genera (Fig. S3), resulting in over 70,000 data points.

### Molecular phenotyping

**Figure 2:**
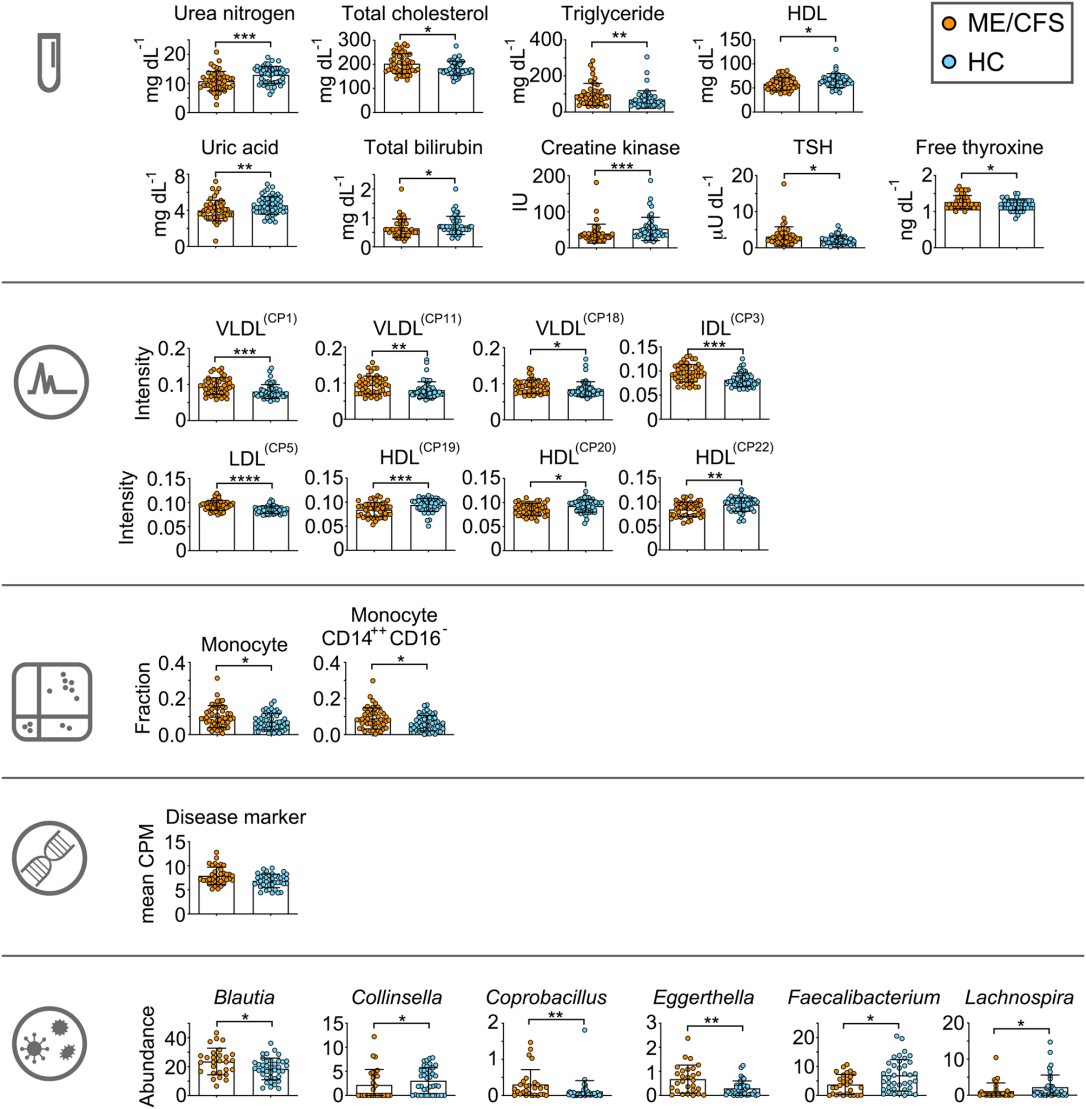
Molecular markers of ME/CFS. Top 26 molecular markers of myalgic encephalomyelitis/chronic fatigue syndrome (ME/CFS) across five platforms (from the top: clinical lab tests, metabolome, immunophenotype, transcriptome, microbiome). For transcriptome data, gene sets with significant difference between ME/CFS patients and healthy controls (HC) (Fig. S4) are represented with geometric mean of the gene set expression level for illustrative purpose. *P* values were determined by two-tailed Mann–Whitney U-test. *P < 0.05, **P < 0.01, ***P < 0.001, ****P < 0.0001. *P*-values were corrected for multiple testing using Benjamini–Hochberg false discovery rate (FDR) method after FDR adjustment at 0.20. *P* values for transcriptome data using Gene Set Enrichment Analysis (GSEA) are indicated in Fig. S4. The number of ME/CFS patients and controls for each platform are summarized in Fig. S1.

We first searched for molecular differences between ME/CFS patients and healthy controls for each of the platforms (Fig. 2). We identified differences in nine blood-based clinical laboratory tests (Fig. 2, Table S1), eight lipoprotein fractions (Fig. 2, Table S3), two immune cell types (Fig. 2, Fig. S2), and six different bacterial genera (Fig. 2, Fig. S3A) after adjusting for false discovery rate to 0.20 for each of the molecular platform. For microbiome analysis, alpha diversity did not significantly differ between patients and controls (Fig. S3B).

For whole blood transcriptomics, given that the expression level of individual genes is subject to technical and biological noise, we searched for sets of genes that were differentially expressed between patients and controls. This method provides greater statistical power and ease of biological interpretation as gene sets are defined using prior knowledge of pathways^38,39^. We used gene set annotations from pathway databases as well as list of genes that were differentially expressed in five prior studies of ME/CFS^8,9,40–42^. One gene set showed significant increase in ME/CFS patients compared to controls after adjusting for false discovery rate to 0.20 (Fig. S4). This gene set was previously found to be upregulated in chronic fatigue syndrome patients^41^.

Among the 26 molecular differences we identified, several of the markers were also discovered in other studies of ME/CFS. These included a decrease in uric acid^12^ and HDL^43^, and an increase in triglyceride^13,43^ for ME/CFS patients. An increase in monocyte number in ME/CFS patients were also identified in one study^21^ although several other studies did not find such difference^44,45^. Additionally, a decrease in *Faecalibacterium*^46–48^ and an increase in *Coprobacillus*^46,48^ were found in ME/CFS patients in previous studies as well as in our current study. Overall, we identified 26 potential markers of ME/CFS from five different molecular platforms including those identified in prior studies.

### Multi-marker analysis

**Figure 3:**
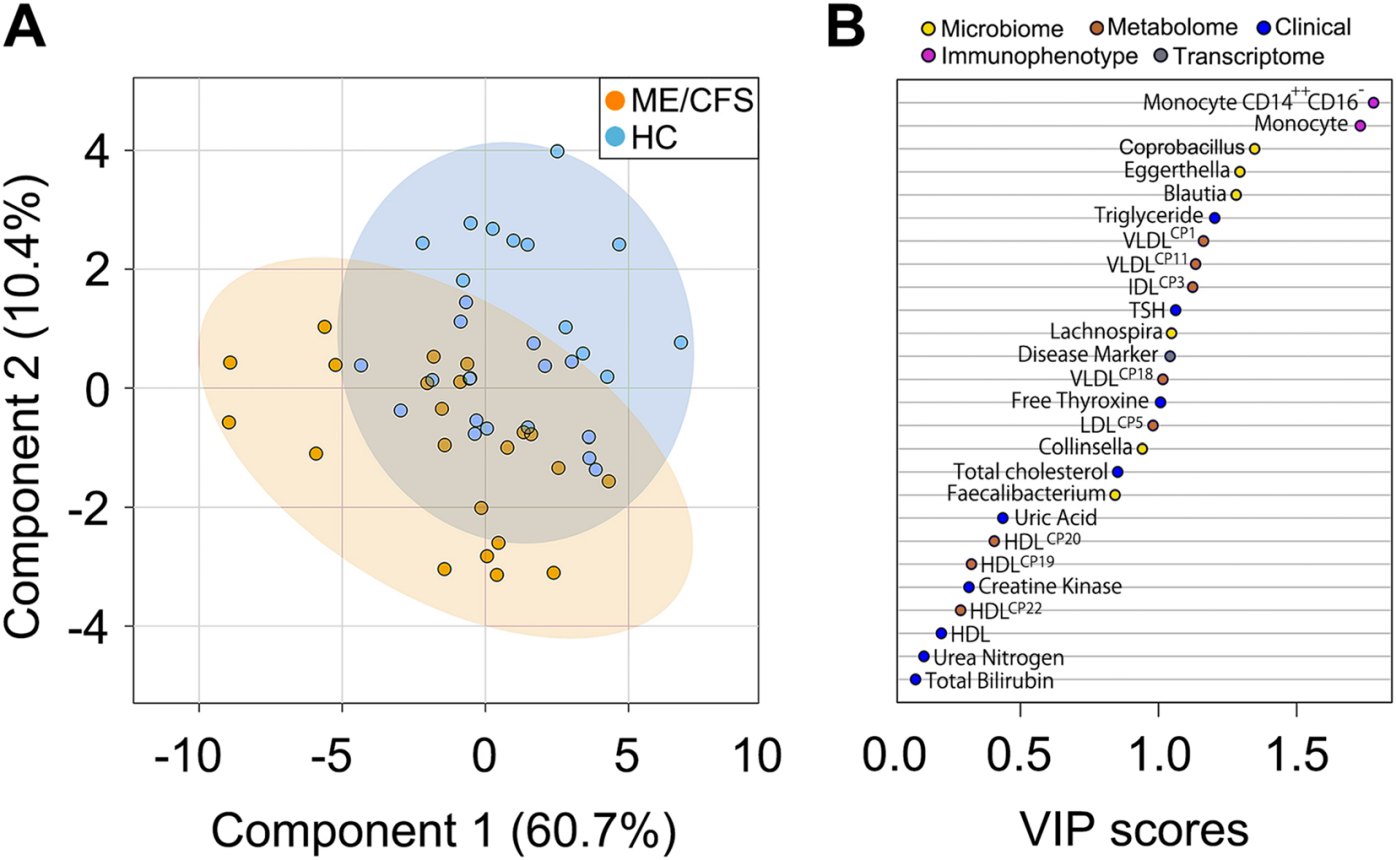
Combinatorial analysis of molecular markers. Combination of top 26 molecular markers for distinguishing myalgic encephalomyelitis/chronic fatigue syndrome (ME/CFS) patients from healthy controls (HC). (**A**) Partial least squares discriminant analysis (PLS-DA) of top 26 molecular markers. (**B**) Variable importance of projection (VIP) scores for distinguishing ME/CFS patients from HC based on component 1. ME/CFS patients (n = 22) and HC (n = 29) with complete molecular profiling across five platforms (clinical lab tests, metabolome, immunophenotype, transcriptome, microbiome) were used for the analysis.

We next examined whether a combination of 26 markers can help distinguish ME/CFS patients from healthy controls. We used partial least square discriminatory analysis (PLS-DA) to project maximum separation between patients and controls using our 26 input markers. We found that while a subset of patients can be separated from controls (Fig. 3A), about half of the patients could not be separated from controls suggesting limitations of our molecular profiling platforms or limitations of biological signals present in peripheral blood or fecal samples.

We next searched for molecular markers that best separated patients from controls using variable importance in projection (VIP) analysis (Fig. 3B). VIP scores estimate the importance of each marker in the PLS-DA projection. Among the 26 markers we identified, monocyte number, abundance of *Coprobacillus*, *Eggerthella*, and *Blautia*, and levels of triglyceride and VLDL performed best in distinguishing patients from controls (Fig. 3B). In particular, three out of six markers from microbiome analysis appeared at the top of the list. This may result from some of the bacterial genera being almost undetectable in one group but present in another group, thus giving rise to a robust separation between patients and controls. Overall, we found that while patients and controls could not be completely separated using our 26 molecular markers, the top markers came from immunophenotype, microbiome, and metabolomic platforms.

### Non-molecular measurements of fatigue

In order to understand the relationship between fatigue and our 26 molecular markers, we quantified fatigue by Chalder fatigue scale, a questionnaire-based method that measures both physical and psychological fatigue^35^. We also assessed quality of sleep using questionnaire-based instrument, PSQIG score^36^. As expected, questionnaire-based assessment of fatigue and sleep quality showed the largest separation between patients and controls (Fig. S5), much more so than any of the molecular and non-molecular measurements we made. This is likely because ME/CFS is diagnosed based on self-reported symptoms of fatigue including unrefreshing sleep. To more objectively and quantitatively assess symptoms related to ME/CFS, we measured patterns of sleep and physical activity using actigraphy^37^. Among the four main measurements from actigraphy, we found the largest difference in number of awakening during total sleep and total sleep time within an average 24 h period (Fig. S5). To quantify cognitive performance, we administered a mathematical test consisting of simple addition of two numbers. We observed a difference in average time to solve math problem but not for percent of correct answer (Fig. S5). We therefore selected these measurements of fatigue-related parameters in our subsequent correlation analysis.

### Correlation network

**Figure 4:**
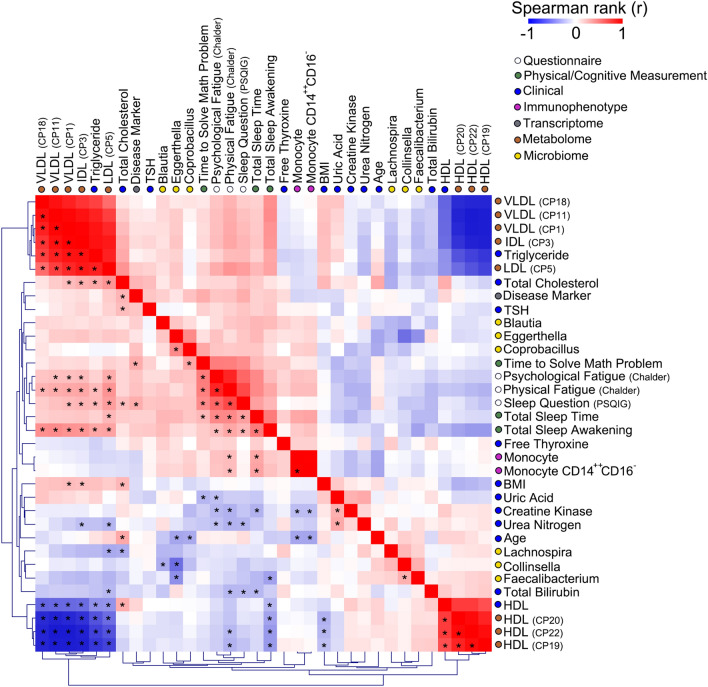
Correlation between top markers of ME/CFS. Blue and red colors indicate Spearman rank correlation value between a pair of markers. Stars (*) denote Spearman rank *P*-value of *P* < 0.05 after adjusting for multiple hypothesis testing using Benjamini–Hochberg false discovery rate (FDR) method set at FDR of 0.05. Circles denote type of data and molecular platform used. Correlation values were clustered using average linkage hierarchical clustering. The number of samples available for a given pair of measurement platforms, as described in Fig. S1, were used for the analysis.

To understand the relationships between fatigue-related measurements and our 26 molecular markers, we performed Spearman rank correlation analysis (Fig. 4). We uncovered some of the expected correlations including positive correlation between triglyceride (clinical laboratory test) and LDL (metabolomics), negative correlation between HDL and LDL, and positive correlation between HDL from clinical laboratory test and HDL from ^1^H-NMR metabolomics. We also identified strong positive correlation between *Coprobacillus* and *Eggerthella* from the microbiome platform. Therefore, a handful of our 26 molecular markers captured overlapping information rather than 26 distinct sets of information. We also observed strong positive correlations between questionnaire-based scores (Chalder, PSQIG) and measures of sleep (total sleep awakening, total sleep time) and cognitive performance (time to solve math problem) (Fig. 4). This suggested that self-assessment of fatigue and sleep quality were reflected in quantitative measures of sleep and cognitive performance using actigraphy and simple math tests.

**Figure 5:**
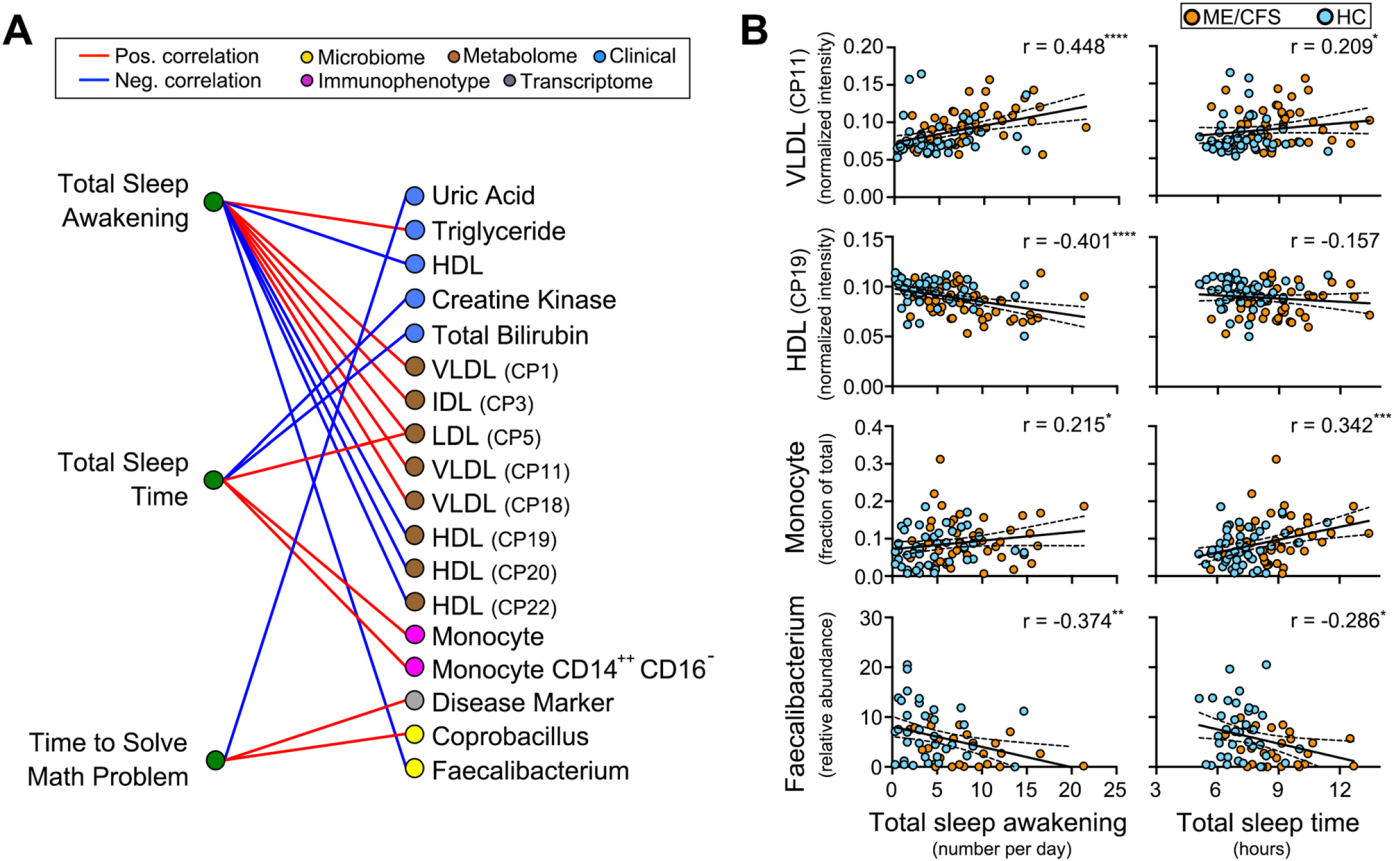
Correlation between measured phenotypes and molecular markers of ME/CFS. (**A**) Spearman rank correlation between three measures related to fatigue (total sleep awakening, total sleep time, time to solve math problem) and molecular markers. Spearman rank correlation with *P* < 0.05 are indicated with red (positive correlation) or blue line (negative correlation). (**B**) Pairwise plot of sleep parameters versus molecular markers. Solid lines are regression lines and dotted lines are 95% confidence interval for the slope. Spearman rank correlation value (r) and corresponding *P* values are indicated. *P < 0.05, **P < 0.01, ***P < 0.001, ****P < 0.0001. *P*-values were corrected for multiple testing using Benjamini–Hochberg false discovery rate (FDR) method after FDR adjustment at 0.20. The number of samples available for a given pair of measurement platforms, as described in Fig. S1, were used for the analysis.

Next, we asked which of the 26 molecular markers more closely correlated with measurements of sleep and cognitive performance (Fig. 5A). These two measures represent more objective assessment of symptoms associated with ME/CFS compared to questionnaire-based measures of sleep quality and psychological fatigue. We found that lipoprotein levels most closely correlated with number of awakening during sleep while monocyte number most closely correlated with the length of sleep (Fig. 5B). These results suggested that different molecular markers were associated with different aspects of sleep. Prior studies have shown that sleep apnea or insufficient sleep were associated with decreased HDL level^49,50^ suggesting that lipoprotein levels may reflect patterns of sleep disruption. We also observed strong negative correlation between *Faecalibacterium* abundance and number of awakening during sleep (Fig. 5B). For cognitive performance (time to solve math problems), a different set of markers showed strong correlation compared to those correlated with sleep. These included *Coprobacillus* and gene set from prior chronic fatigue syndrome study. These results suggested that lipoprotein levels, monocyte number, and *Faecalibacterium* abundance may reflect sleep-related changes in ME/CFS patients.

### Medication and duration of illness

We examined whether any of the 26 molecular markers in our study were affected by the use of medications. Among the ME/CFS patients with information on medication, those taking antidepressants (n = 9) showed no significant difference in 25 of the molecular markers when compared to patients not taking antidepressants (n = 36) (Mann–Whitney U-test; *P* > 0.05). The exception was a gene set that was previously elevated in chronic fatigue syndrome (CFS) patients (Fig. S6A). ME/CFS patients on antidepressants, when compared to patients not on antidepressants, showed higher expression level of the CFS marker gene set. Given that the original study in which the gene set was derived allowed patients to continue taking their prescribed medication^45^, the gene set may reflect the use of antidepressants among ME/CFS patients rather than the changes resulting from the syndrome itself. Antidepressant use did not affect sleep parameters or cognitive performance (Fig. S6B). We also found that ME/CFS patients on sleeping pill (n = 9) showed lower total cholesterol level compared to patients not taking sleeping pill (n = 36) (Fig. S6C). Interestingly, the actigraphy measurement of sleep did not differ with the use of sleeping pill suggesting that the medication did not improve abnormal sleep but only restored total cholesterol level to healthy control level. We did not observe differences in the remaining 25 molecular markers based on the use of sleeping pill. We note that our analysis of medication use is limited as the number of patients on each medication was few.

To assess the diagnostic potential of our molecular markers, we examined whether our 26 molecular markers still showed significant difference during short-duration of ME/CFS. We separated patients into two groups (≤ 3 years, > 3 years) similar to prior biomarker study of ME/CFS^18^. We found that lipoprotein markers as well as sleep and cognitive measurements were already different during short-duration (≤ 3 years) of ME/CFS and were still different during long-duration (> 3 years) of illness (Fig. S7). We also found that microbiome changes were much more prominent during the short-duration of illness (≤ 3 years) compared to long-duration of illness (> 3 years) suggesting that microbiome markers may reflect temporary changes during the early phase of ME/CFS (Fig. S7). For monocyte number, we did not observe significant changes during the short-duration of illness (≤ 3 years). Overall, we found that sleep, lipoprotein, and microbiome changes occurred during the early course of illness, an important criterion for future biomarker application.

## Discussion

To gain insights into molecular markers that best distinguish myalgic encephalomyelitis/chronic fatigue syndrome (ME/CFS) patients from healthy controls, we performed deep phenotyping of cases and controls using five molecular platforms, sleep and cognitive measurements, and questionnaire-based assessment of fatigue. Our analysis identified 26 potential molecular markers of ME/CFS. Among these, monocyte number, microbiome profiles, and lipoprotein profiles provided the highest information value in distinguishing patients from controls. Although these 26 markers could not completely separate patients from controls, a subset of these molecular platforms such as lipoprotein profiles and microbiome analysis, showed difference even during the early phases of illness (≤ 3 years). These platforms can be extended to a larger cohort with longitudinal design to assess the diagnostic performance of markers. Given that lipoprotein profiling is part of a routine clinical test and that microbiome analysis is starting to be offered as a diagnostics service, these two platforms may be a promising diagnostic tool for ME/CFS.

One of the key advantages of deep phenotyping is that we can now start to uncover relationships between markers across platforms to gain insights into the syndrome. We found that self-assessment of fatigue (Chalder fatigue scale) was most closely correlated with more objective measurements of sleep (actigraphy) and cognitive performance (simple mathematical test). Interestingly, some of our top markers including elevated LDL were more closely correlated with sleep abnormality (sleep awakening) compared to cognitive performance. Previous study has shown that statin therapy, which is used to lower LDL level, is associated with decreased number of awakening during sleep^51^ suggesting that there may be a mechanistic link between sleep and lipoprotein profile. With unrefreshing sleep being one of the symptoms and major complaints of ME/CFS patients, molecular markers that reflect sleep or altered circadian rhythm^52^ may serve as additional markers of ME/CFS. One note of caution is that the mathematical test in our study is an unvalidated tool and future study should include validated neuropsychological tools in correlating molecular changes to cognitive measurements. 

In relation to past studies, we uncovered several molecular markers that were previously reported in ME/CFS patients. These include decreased uric acid^12^, HDL^43^, and *Faecalibacterium*^46–48^, and increased triglyceride^13,43^, monocyte number^21^, and *Coprobacillus*^46,48^. However, there were also markers that were not identified in our study. Aside from the differences in cohort, these differences could result from the differences in the technological platforms and source of biological samples used in our study. One main difference is we used whole blood instead of PBMC for transcriptome analysis which could elevate the contribution of non-immune cell, mainly red blood cells, to the gene expression profile. Another difference is we used ^1^H-NMR for metabolomics while many other studies use GC–MS, LC–MS, or CE–MS method. More studies are needed to assess the impact of technological platforms in detecting some of these reported markers.

One of the main limitations of our study is that some of the markers we identified reflect comorbidity associated with ME/CFS rather than ME/CFS itself. In fact, several of the markers identified in our study are also markers of major depressive disorder including decreased uric acid, urea nitrogen, and total bilirubin^53^, and increased monocyte number^54^. Given that patients suffering from ME/CFS can sometimes develop depression as a result of unresolved debilitating fatigue^55^, it is possible that some of the markers captured comorbid depression. However, molecular markers that were different during the early phase of illness (≤ 3 years), such as decreased HDL, were not part of markers previously identified in major depressive disorder. HDL is elevated in patients with major depressive disorder^53^ while it is decreased in our ME/CFS cohort. It is important to note that depression which results from ME/CFS does not resolve with antidepressant fluoxetine and may represent a different form of depression than those of major depressive disorder^56^. Since the ME/CFS patients who joined the present study did not show a typical depression, future study of these markers in a larger cohort should include a considerable number of patients with comorbid depression to assess the specificity of our markers for ME/CFS.

Another important comorbidity to note regarding our study is that ME/CFS is often accompanied by irritable bowel syndrome, which alters the composition of gut microbiota. Although we observed changes in the microbiome profile during the early phase of illness (≤ 3 years), a previous study found that a decrease in *Faecalibacterium* was a top marker for ME/CFS with irritable bowel syndrome while a decrease in *Bacteroides vulgatus* was a top biomarker for ME/CFS without irritable bowel syndrome^48^. Therefore, several of the markers from our microbiome analysis may also reflect irritable bowel syndrome comorbidity. Future study of our microbiome markers should include a larger cohort with assessment of irritable bowel syndrome.

ME/CFS represents a collection of heterogeneous illness with diagnostic criteria meant to capture patients at the extreme end of the fatiguing illness^5,23^. Our deep phenotyping of ME/CFS in Japanese cohort revealed several molecular markers of illness that strongly correlated with sleep abnormality. Given that ME/CFS spans multiple symptoms^1,2,23^, some of the molecular markers identified in our study may reflect a subset of symptoms that were more prevalent in our cohort. This could influence whether potential biomarkers identified in our cohort can be applied to another cohort for ME/CFS. Although it remains unclear whether sleep abnormality is an over-represented symptom in our Japanese cohort compared to other cohorts, these molecular markers can still serve as measures of sleep-related changes in ME/CFS patients. Patients positive for these sleep-related molecular markers can then be recommended for more extensive analysis of sleep using actigraphy or polysomnography to objectively assess symptom related to unrefreshing sleep. In addition, with our extensive knowledge through PET molecular imaging and neurofunctional imaging^23–34^, the functional and molecular abnormality in the brain could be connected with these peripheral molecular markers. In our on-going studies, we have started the correlation studies among brain dysfunction, neuroinflammation detected by PET, and these molecular markers. Future studies that quantitatively measure symptoms related to fatigue and functional deterioration, including cognitive dysfunction and unrefreshing sleep, can help clarify the relationship between newly identified molecular markers and ME/CFS. These in turn could help untangle heterogeneity inherent in ME/CFS, which could aid in tailoring potential therapeutics for patient subgroups.

## Materials and methods

### Study design

Study subjects included 48 myalgic encephalomyelitis/chronic fatigue syndrome (ME/CFS) patients and 52 healthy controls, recruited at the Osaka City University Hospital Fatigue Clinical Center (Osaka, Japan). The study was approved by the ethics committees of Osaka City University Graduate School of Medicine (Approval No. 1498, 2151, 1499), and of RIKEN (Approval No. KOBE-IRB-11-13, YOKOHAMA-IRB-H24-17), and was conducted in accordance with the Declaration of Helsinki. All subjects, ME/CFS patients (n = 48) and healthy individuals (n = 52), provided written informed consent for participation in the study before enrolment. Healthy individuals were confirmed not to have abnormal results on any major clinical laboratory tests (hemoglobin, CRP, albumin, triglycerides, glucose, AST, ALT, or cholesterol, etc.), not to be in the BMI range of ≥ 30 or < 17, not to have subjective sleep problems, problems in daily life by fatigue, or be a shift worker. ME/CFS patients who visited the outpatient clinic of Osaka City University Hospital were randomly enrolled into the study. ME/CFS patients were diagnosed based on meeting both the 1994 Center for Disease Control clinical criteria (Fukuda criteria)^1^ and the International Consensus Criteria^2^ by the specialists at the Osaka City University Hospital. All patients also fulfilled the diagnostic criteria of Systemic Exertion Intolerance Disease (SEID, 29). All subjects were non-smokers. Degree of physical and psychological fatigue were assessed using Chalder fatigue scale^35^. The quality of sleep was assessed using Pittsburgh Sleep Quality Index Global (PSQIG) score^36^. Chalder fatigue scale and PSQIG were not used as part of the diagnosis process.

### Sample collection and processing

Case and control subjects were fasted after 9:00 p.m. of the last night of the clinical test day (for at least 12 h) and peripheral blood was drawn between 9:00 am and 12:00 p.m. and collected into EDTA blood collection tubes. Blood samples were analyzed on automatic biochemical analyzer for biochemical parameters listed in Table S1. Whole blood was used for transcriptome analysis using Cap Analysis of Gene Expression (CAGE). For FACS and metabolome analysis, 5 mL of blood was diluted with 30 mL of Dulbecco′s Phosphate Buffered Saline (D-PBS) in 50 mL Falcon tube and 15 mL of Ficoll Paque PLUS (GE Healthcare) were gently added, then centrifuged for 35 min at 400 × *g* at 20 °C. Top layer (plasma) was transferred to a new tube for metabolomics. For immunophenotype analysis, the middle layer containing lymphocytes and monocytes were transferred to a new 50 mL Falcon tube, filled up to 50 mL with D-PBS, centrifuged at 300 × *g* for 10 min at 20 °C. Supernatant was removed and the tube was refilled with D-PBS and centrifugation step was repeated. The resulting pellet was resuspended with 1 mL of 2% fetal calf serum (FCS)/D-PBS for antibody staining.

### Actigraphy

Activity and sleep patterns were monitored using actigraphy method^57^. Activity was monitored in ME/CFS patients for seven days and healthy controls for three days by having subjects wear ActiGraph (Ambulatory Monitoring, Inc., USA) on non-dominant hand. The actigraph software using Cole–Kripke algorithm^58^ was used to calculate number of awakening during total sleep, total sleep time, total sleep efficiency, and total activity time within an average 24 h period.

### Simple mathematical tests

Simple mathematical tasks consisting of addition of two single digit numbers were administered to case and control subjects for 5 min. Percentage of total correct answers and average time to solve mathematical problems were measured.

### Immunophenotype

To 50 μL of lymphocytes and monocytes in 2% FCS/D-PBS, antibodies (Table S4) were added at the indicated volume for 30 min at 4 °C. 1 mL of 2% FCS/D-PBS were added to stained cells, centrifuged at 1200 rpm for 5 min at 4 °C, washed with 2% FCS/D-PBS twice, and resuspended in 300 μL of 2% FCS/D-PBS, and analyzed using FACS Aria III (BD Bioscience). The gating strategy is shown in Fig. S8. Fraction of cells within each gating scheme was used for subsequent analysis.

### Transcriptome analysis

RNA from whole blood was obtained using Ribopure blood kit (Ambion). CAGE libraries were prepared as described previously^59^. CAGE libraries were sequenced with the 50 bases single-end mode on the Illumina HiSeq 2500 platform according to the manufacturer’s instructions (Illumina). The raw reads were processed in MOIRAI pipeline (Version 20121120)^60^ system as follows: ligation adaptor sequences were trimmed; rRNA-derived reads and a base called 'N' were discarded by rRNAdust program, the processed reads were aligned to human reference genome (hg19) using BWA (Version: 0.7.10-r789)^61^, poorly mapped reads (mapping quality < 20) were discarded using SAMtools (Version: 0.1.18). The robust TSS sets identified in the FANTOM5 project were used as TSS reference, and the count of 5ʹ-end of remaining CAGE reads mapped on TSS regions were used as raw signal of promoter expression. The expression signals were normalized by relative log expression method in the edgeR package^62^. Promoter expression with at least 1 cpm for all subjects were selected for subsequent analysis. Gene set enrichment analysis was performed as described before^39^ using gene set annotation from curated pathways (v7.0: KEGG, BioCarta, Reactome). To obtain list of genes that were differentially expressed in previous studies of ME/CFS, we obtained gene list from publication^8,41^, or obtained dataset from NCBI GEO (GSE98139, GSE16059, GSE14577)^9,40,42^. For the three datasets from NCBI GEO, we filtered out genes that did not meet our filtering criteria. The criteria were normalized count of > 0 for GSE98139, expression value of > 6.6 (corresponding to Affymetrix expression value of 100) for GSE16059, and expression value of > 100 for GSE14577 for all samples within the dataset. Signal-to-noise ratios were calculated between cases and controls and top 100 up- and down-regulated genes were obtained as gene set. For GSEA, samples were permuted 1000 times using signal-to-noise ratio ranking to estimate *P*-value. For genes with multiple promoters, promoter with highest expression value was selected so that one gene corresponded to one gene expression (promoter) data. For correlation analysis, geometric mean for genes assigned as “core enrichment” from GSEA were calculated to obtain a single numerical representation of gene expression levels within a gene set.

### Metabolome analysis

For metabolome analysis, 50 μL of deuterium oxide containing 5 mM DSS-d_6_ reference material (Wako) was added to 450 μL of plasma. Prepared samples were measured on an NMR spectrometer (Bruker Avance II 700; Bruker Biospin) at 298 K and ^1^H-NMR was measured using a Bruker standard program (noesypr1d) with 32,768 data points, 32 scans, 4 dummy scans, a 16-ppm spectral width, and 2 s relaxation delay as described^63^. Annotation of signals were made with two-dimensional *J*-resolved NMR measurement using Bruker standard program (jresgpprgf) with 32 data points for F1 and 16,384 data points for F2, 16 scans, 16 dummy scans, a 50-Hz spectral width for F1, a 18-ppm spectral width for F2, and a 2 s relaxation delay as described^64^. For two-dimensional *J*-resolved NMR measurements, intensity values were normalized to total intensity, both across subjects and metabolites. Signals were annotated with SpinCouple program^65^ with reference to the Human Metabolome Database^66^. For annotation of lipoproteins, the diffusion-edited pulse program (ledbpgppr2s1d) was conducted with 66,560 data points, 64 scans, 4 dummy scans, a 16-ppm spectral width, and 1 s relaxation delay. Then the diffusion-edited spectra were divided into 30 fractions in the –CH_3_ regions^67^ and fraction corresponding to HDL was assigned based on correlation to the clinical laboratory-based test result of HDL value using peak assignment in Table S3.

### Fecal microbiome analysis

Stool specimens were collected and sent to Osaka City University Hospital from the patients and healthy volunteers and stored at − 20 °C until the transfer of all fecal samples to RIKEN Yokohama Institute. DNA extraction, 16S rRNA sequencing, and analysis were performed as described before^68^. Freeze-dried fecal samples were suspended in 10% sodium dodecyl sulfate, 10 mM Tris–HCl, and 1 mM EDTA (pH 8.0), then disrupted with 0.1-mm zirconia/silica beads (BioSpec Products) by shaking at 1500 rpm for 10 min. After centrifugation, bacterial DNA was purified using 25:24:1 phenol–chloroform–isoamyl alcohol and precipitated by ethanol and sodium acetate. The resulting DNA was treated with RNase A and precipitated by polyethylene glycol. The V1–V2 variable region of the 16S rRNA was PCR amplified using 27Fmod-338R primer pairs for 22 cycles, indexed using Nextera XT index primers, and sequenced on MiSeq (Illumina). 16S rRNA sequencing data were processed using QIIME software package^69^. An operational taxonomic unit (OTU) was defined at 97% similarity. OTU relative abundance below 0.05% were filtered to remove noise. OTU taxonomy was assigned based on comparison to Greengenes Database using RDP classifier with confidence level set at 0.5^70^.

### Statistical analysis

Differences between ME/CFS patients and healthy controls were analyzed using Mann–Whitney U test (GraphPad Prism) and corrected for multiple testing using Benjamini–Hochberg correction method^71^. For comparison of three or more sample groups, Kruskal–Wallis test was performed followed by Dunn’s multiple comparison post-test (GraphPad Prism). Power calculation was not performed prior to the initiation of the study as accepted methods to assess statistical power across multiple omics measurements did not exist at the time. For correlation between markers, Spearman rank correlation were calculated (GraphPad Prism) and markers were clustered based on similarity in correlation value using average linkage hierarchical clustering. For multi-marker analysis, data were log transformed and normalized by sample median, and auto scaled. Separation between ME/CFS patients and healthy controls were visualized with partial least squares discriminant analysis (PLSDA) and importance of markers for separating the two groups were evaluated using variable importance in projection (VIP) score. Multi-marker analysis were performed using MetaboAnalyst 4.0^72^.

## Supplementary information

Supplementary information.

## Data Availability

All data associated with this study are presented in the paper or the Supplementary Information.
