# Supplementary material for: Deep phenotyping of myalgic encephalomyelitis/chronic fatigue syndrome in Japanese population
Source: Sci Rep. 2020 Nov 16;10:19933. doi: 10.1038/s41598-020-77105-y (PMC7669873; doi:10.1038/s41598-020-77105-y)
Supplement: Supplementary file 1 — Supplementary information. [file 41598_2020_77105_MOESM1_ESM.pdf]

Supplementary Information

Title: Deep Phenotyping of Myalgic Encephalomyelitis/Chronic Fatigue Syndrome in Japanese Population

Authors: Toshimori Kitami[1]†*, Sanae Fukuda[2-4]†, Tamotsu Kato[1]†, Kouzi Yamaguti[2], Yasuhito Nakatomi[2,5], Emi Yamano[2,4,6], Yosky Kataoka[2,4,6-7], Kei Mizuno[2,4,6], Yuuri Tsuboi[8],Yasushi Kogo[9], Harukazu Suzuki[1], Masayoshi Itoh[9], Masaki Suimye Morioka[1], Hideya Kawaji[1,9], Haruhiko Koseki[1], Jun Kikuchi[8,10-11]‡, Yoshihide Hayashizaki[9]‡, Hiroshi Ohno[1,11]‡, Hirohiko Kuratsune[2-6]‡, Yasuyoshi Watanabe[2,4,6]‡*

Affiliations:

[1]RIKEN Center for Integrative Medical Sciences, Kanagawa, Japan.

[2]Osaka City University Graduate School of Medicine, Osaka, Japan.

[3]Kansai University of Welfare Sciences, Osaka, Japan.

[4]RIKEN Center for Biosystems Dynamics Research, Hyogo, Japan.

[5]Nakatomi Fatigue Care Clinic, Osaka, Japan.

[6]RIKEN Compass to Healthy Life Research Complex Program, Hyogo, Japan.

[7]RIKEN Baton Zone Project, RIKEN-JEOL Collaboration Center, Hyogo, Japan.

[8]RIKEN Center for Sustainable Resource Sciences, Kanagawa, Japan.

[9]RIKEN Preventive Medicine and Diagnosis Innovation Program, Saitama, Japan.

[10]Graduate School of Bioagricultural Sciences, Nagoya University, Aichi, Japan.

[11]Graduate School of Medical and Life Sciences, Yokohama City University, Kanagawa, Japan.

*Corresponding authors. Email: yywata@riken.jp (Y.W.), toshimori.kitami@riken.jp (T.K.).

†These authors contributed equally to this work.

‡Senior authors.

Supplementary Information

Fig. S1. Data completeness across a panel of markers examined for ME/CFS patients and HC.

Fig. S2. Immunophenotyping of peripheral blood mononuclear cells (PBMCs).

Fig. S3. Fecal microbiome analysis.

Fig. S4. Gene-set enrichment analysis (GSEA) of ME/CFS marker genes.

Fig. S5. Non-molecular measures of fatigue.

Fig. S6. Effect of medication on molecular markers.

Fig. S7. Duration of illness and molecular markers.

Fig. S8. FACS gating strategy for immunophenotype analysis.

Table S1. Baseline characteristics and clinical laboratory results of study cohort.

Table S2. Metabolite profiling of ME/CFS patients and HC.

Table S3. Lipoprotein profiling of ME/CFS patients and HC.

Table S4. List of antibody sets used for FACS analysis in Fig. S2.

Supplementary Materials:

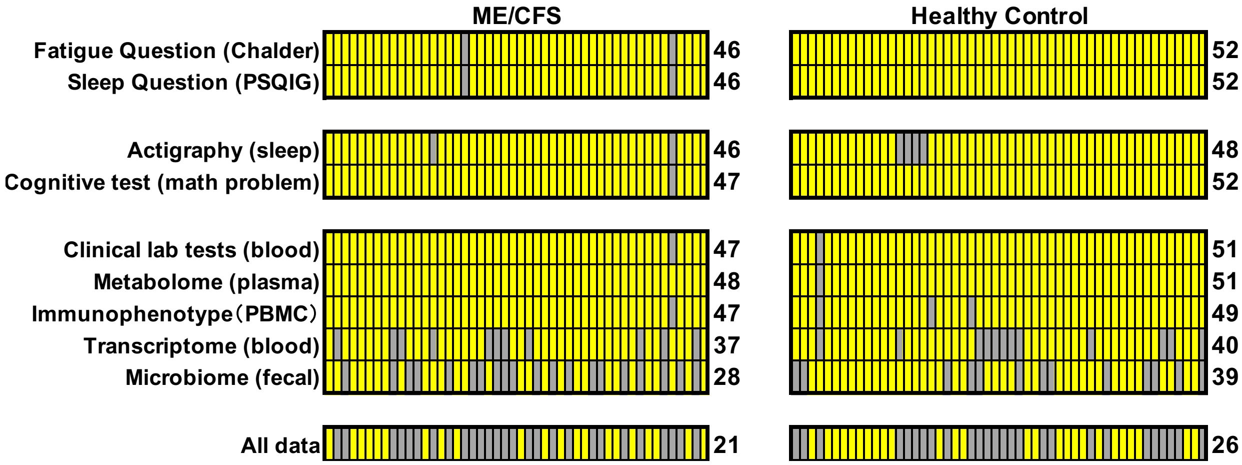

Fig. S1. Data completeness across a panel of markers examined for ME/CFS patients and HC. Each column represents a subject from myalgic encephalomyelitis/chronic fatigue syndrome (ME/CFS) or healthy control (HC). Yellow box indicates presence of data and grey box indicates absence of data. Numbers on the right side indicate the number of datasets for each platform. For comparison within a given platform (Fig. 2, Fig. S1-S7), all available samples (yellow box) within a platform were used for the analysis. For correlation analysis involving comparison between platforms (Fig. 4, 5), the number of samples with both platforms being available (both platforms having yellow boxes) were used. For combinatorial analysis using PLS-DA (Fig. 3), the number of samples with all molecular platforms being available (yellow boxes for clinical lab tests, metabolome, immunophenotype, transcriptome, microbiome) were used.

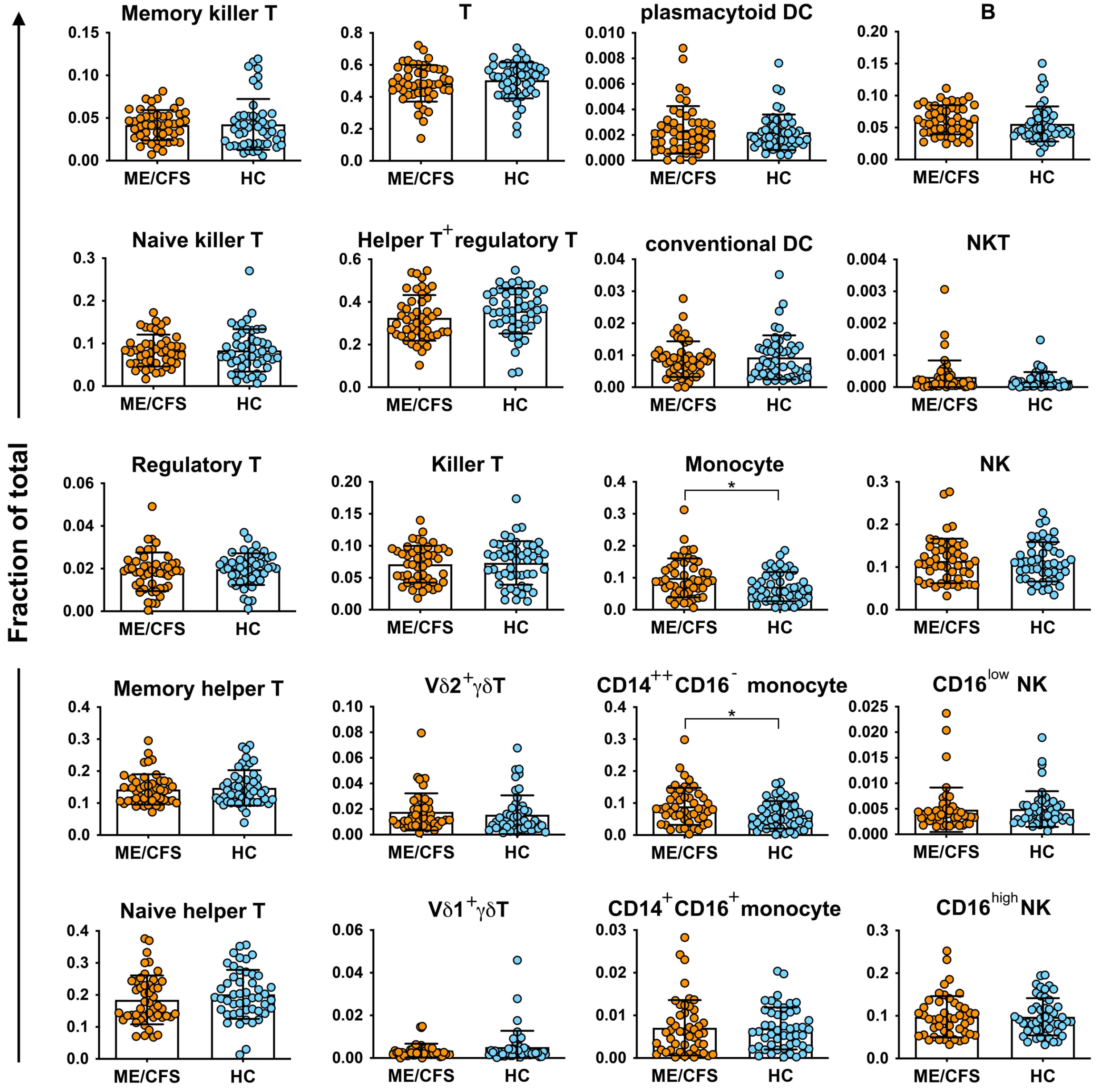

Fig. S2. Immunophenotyping of peripheral blood mononuclear cells (PBMCs). Fraction of each immune cell type in blood of myalgic encephalomyelitis/chronic fatigue syndrome (ME/CFS) patients (n=47) and healthy controls (HC) (n=49). Fraction is based on the amount of cells within a particular antibody staining pattern (total 6 different sets of patterns) using 5mL of starting blood sample, described in Table S4. *P* values were determined by two-tailed Mann-Whitney U-test with Benjamini-Hochberg correction, adjusted for false discovery rate (FDR) of less than 0.20. $^{*}P < 0.05$.

A

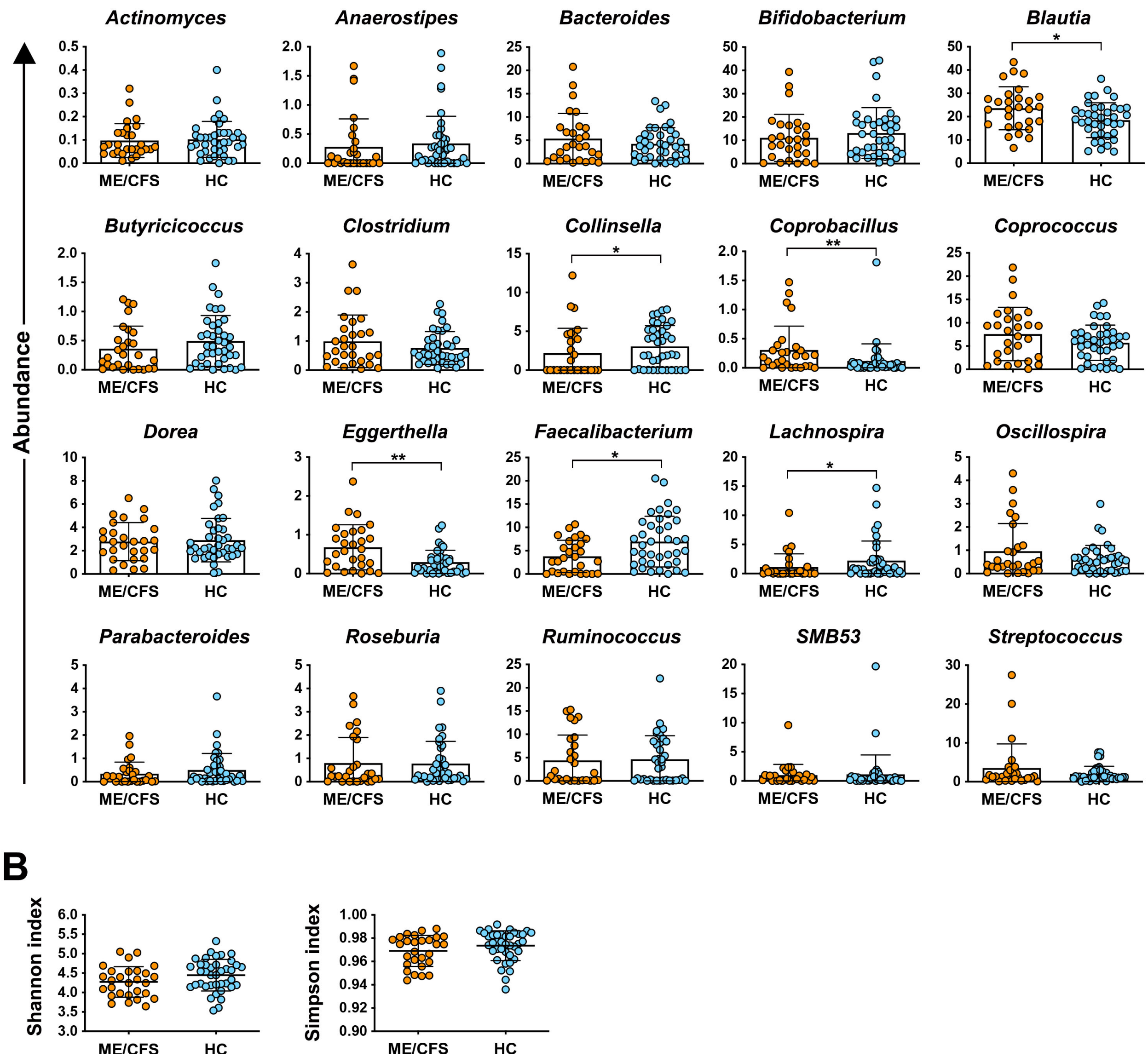

Fig. S3. Fecal microbiome analysis. (**A**) Genus level abundance (median >0.05% cutoff) of bacteria between myalgic encephalomyelitis/chronic fatigue syndrome (ME/CFS) patients (n=28) and healthy controls (HC) (n=39). (**B**) Alpha diversity was determined by Shannon index and Simpson index. *P* values were determined by two-tailed Mann-Whitney U-test with Benjamini-Hochberg correction, adjusted for false discovery rate (FDR) of less than 0.20. *$P < 0.05$, **$P < 0.01$.

A

Disease Markers CFS

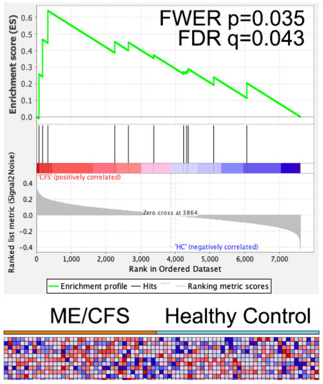

Fig. S4. Gene-set enrichment analysis (GSEA) of ME/CFS marker genes. (**A**) GSEA enrichment plot and the heatmap of chronic fatigue syndrome (CFS) marker gene set (ref. 43) in our patient cohort. False discovery rate (FDR) and family-wise error rate (FWER) adjusted p-value are shown. The number of samples were, ME/CFS (n=37) and healthy control (n=40).

43. S. D. Vernon, E. R. Unger, I. M. Dimulescu, M. Rajeevan, W. C. Reeves, Utility of the blood for gene expression profiling and biomarker discovery in chronic fatigue syndrome. Dis. Markers 18, 193-199 (2002).

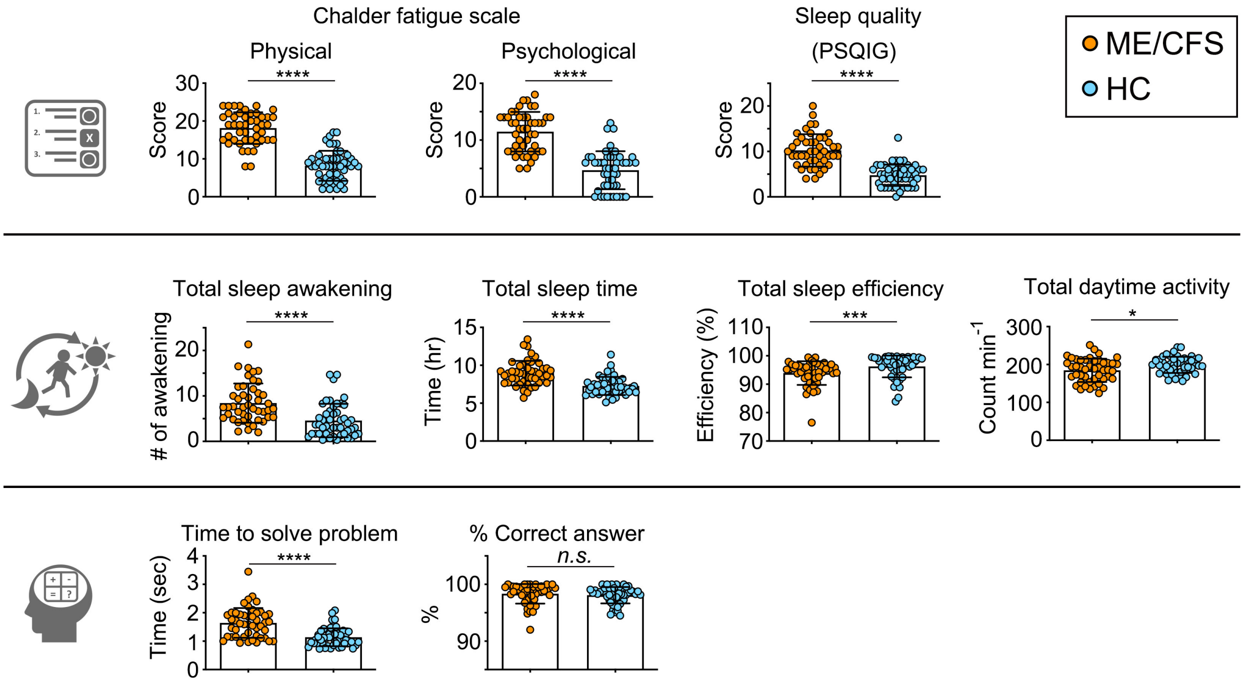

Fig. S5. Non-molecular measures of fatigue. Physical and psychological fatigue was assessed using Chalder fatigue scale, and sleep quality was assessed using Pittsburgh Sleep Quality Index Global (PSQIG) score. Sleep and activity pattern within an average 24 hour period were measured using actigraphy. Cognitive performance was assessed by administration of simple mathematical problems. *P* values were determined by two-tailed Mann-Whitney U-test with Benjamini-Hochberg correction, adjusted for false discovery rate (FDR) of less than 0.20. $^{*}P < 0.05$, $^{**}P < 0.01$, $^{***}P < 0.001$, $^{****}P < 0.0001$, or not significant (*n.s.*). The number of ME/CFS patients and healthy controls (HC) for each platform are summarized in Fig. S1.

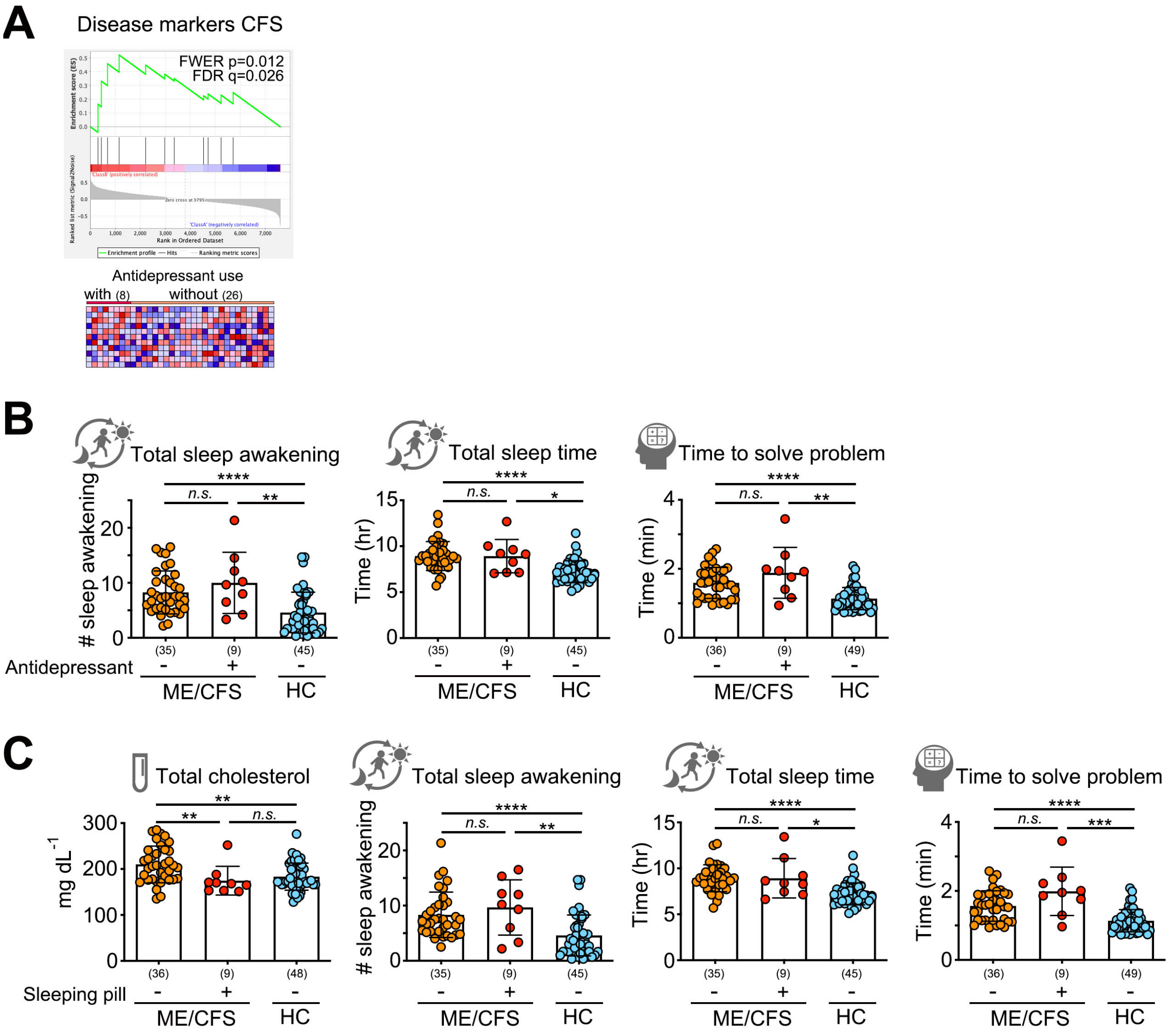

Fig. S6. Effect of medication on molecular markers. (**A**) Effect of antidepressant on transcriptome data. Chronic fatigue syndrome (CFS) marker genes from a published study (ref. 43) were compared between ME/CFS patients taking antidepressant ("with") versus patients not taking antidepressant ("without") using gene-set enrichment analysis (GSEA). False discovery rate (FDR) and family-wise error rate (FWER) adjusted p-value are shown. (**B**) Effect of antidepressant on sleep and cognitive parameters. (**C**) Effect of sleeping pill on total cholesterol, sleep, and cognitive parameters. Sample sizes for each group are indicated in parentheses. *P* values were determined by Kruskal-Wallis test followed by Dunn's multiple comparison test. **P* < 0.05, ***P* < 0.01, ****P* < 0.001, *****P* < 0.0001, or not significant (*n.s.*).

43. S. D. Vernon, E. R. Unger, I. M. Dimulescu, M. Rajeevan, W. C. Reeves, Utility of the blood for gene expression profiling and biomarker discovery in chronic fatigue syndrome. Dis. Markers 18, 193-199 (2002).

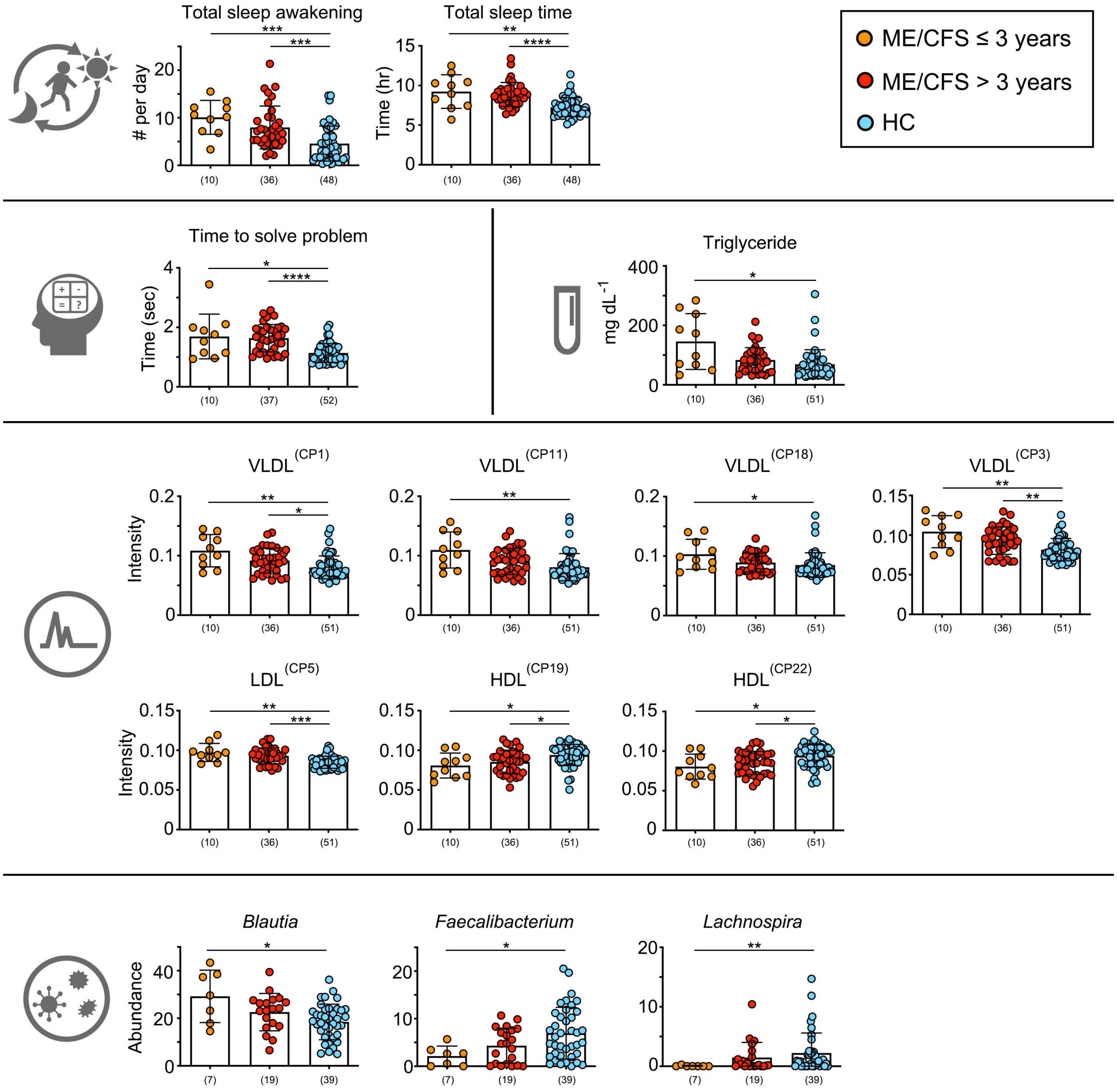

Fig. S7. Duration of illness and molecular markers. Effect of short (≤ 3years) versus long (> 3 years) duration of ME/CFS on molecular markers, sleep measurement, and cognitive performance. Sample sizes for each group are indicated in parentheses. *P* values were determined by Kruskal-Wallis test followed by Dunn's multiple comparison test. **P* < 0.05, ***P* < 0.01, ****P* < 0.001, *****P* < 0.0001.

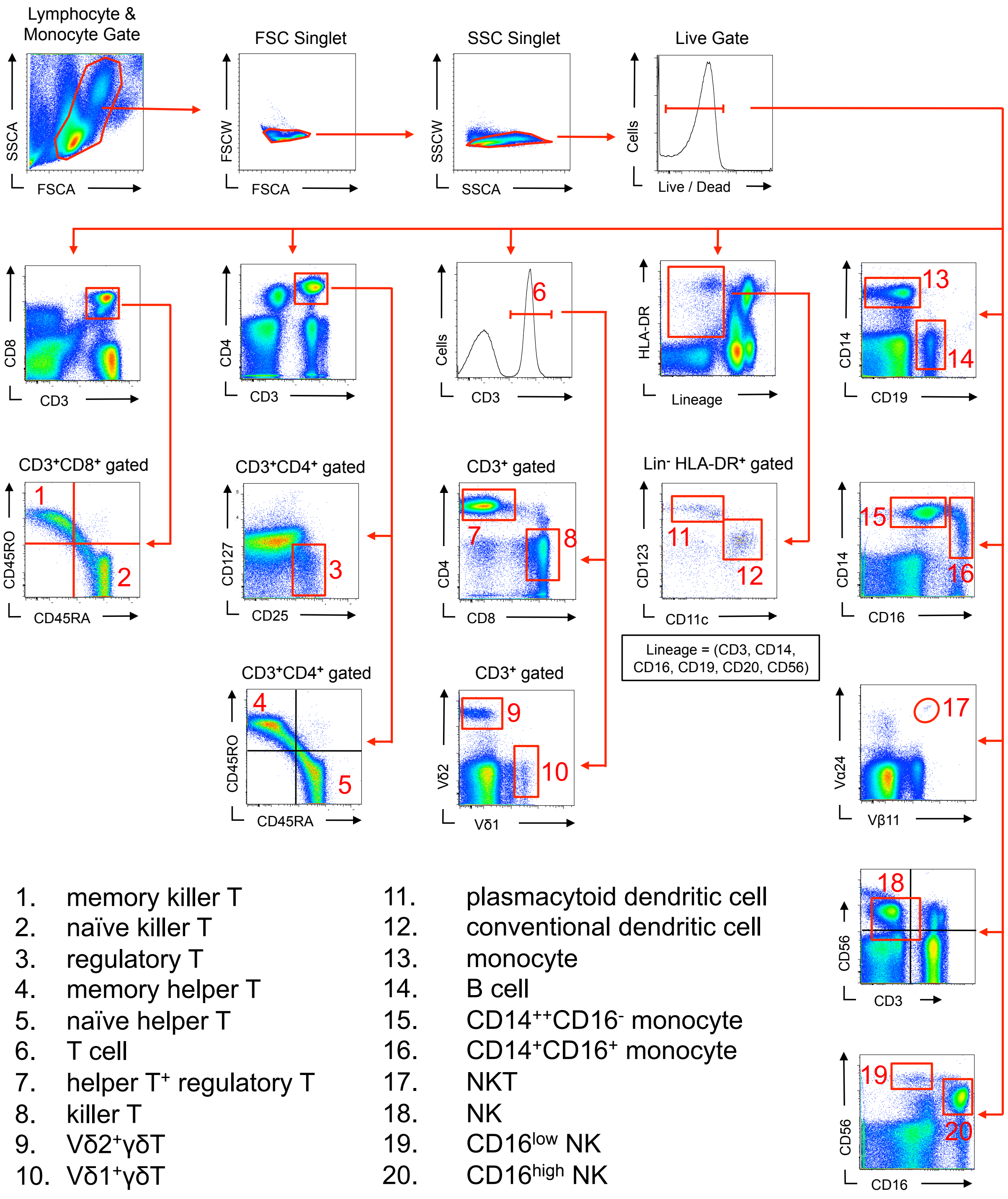

Fig. S8. FACS gating strategy for immunophenotype analysis. Proportion of 20 different types of immune cells was analyzed using FACS with antibodies listed in Table S4.

Table S1. Baseline characteristics and clinical laboratory results of study cohort. Clinical laboratory measurements are represented as median and interquartile range (in parentheses) for continuous variable. *P* values were determined by Fisher's exact test for gender and two-tailed Mann-Whitney U-test for the remaining parameters. *P*-values were corrected for multiple testing using Benjamini-Hochberg false discovery rate (FDR) method after FDR adjustment at 0.20. *P* values are as indicated, or not significant (n.s.).

Abbreviation	Categories	Unit	ME/CFS	HC	*P* value
	Gender (Female/Male)		41F , 6M	47F , 5M	n.s.
	Age	yr	37 (33 - 42)	40 (34 - 45)	n.s.
	BMI	kg per m^2	21.0 (19.0 - 23.0)	20.0 (19.8 - 22.0)	n.s.
WBC	White Blood Cell	x100 per µL	52 (43 - 61)	51 (42 - 60)	n.s.
RBC	Red Blood Cell	x10,000 per µL	436 (421 - 472)	433 (408 - 458)	n.s.
HB	Hemoglobin	g per dL	13.1 (12.5 - 13.9)	13.2 (12.1 - 13.7)	n.s.
HT	Hematocrit	%	38.8 (37.7 - 41.5)	39.6 (36.3 - 40.9)	n.s.
MCV	Mean Corpuscular Volume	fl	90.2 (86.7 - 91.9)	90.7 (87.8 - 92.9)	n.s.
MCH	Mean Corpuscular Haemoglobin	pg	30.4 (29.1 - 30.9)	30.2 (29.2 - 31.1)	n.s.
MCHC	Mean Corpuscular Haemoglobin Concentration	%	33.6 (32.9 - 33.9)	33.3 (32.8 - 33.7)	n.s.
PLT	Platelet	x10,000 per µL	24.2 (20.8 - 29.1)	24.6 (20.4 - 28.1)	n.s.
CRP	C-Reactive Protein	mg per dL	0.02 (0.01 - 0.03)	0.02 (0.01 - 0.03)	n.s.
TP	Total Protein	g per dL	7.2 (6.8 - 7.4)	7.2 (6.9 - 7.4)	n.s.
UN	Urea Nitrogen	mg per dL	10.6 (8.7 - 12.3)	13.6 (10.3 - 15.0)	0.0005
UA	Uric Acid	mg per dL	3.9 (3.3 - 4.5)	4.4 (3.9 - 5.2)	0.0045
CRE	Creatinine	mg per dL	0.64 (0.59 - 0.70)	0.70 (0.59 - 0.76)	n.s.
T-CHO	Total Cholesterol	mg per dL	196 (171 - 239)	174 (166 - 209)	0.0206
TG	Triglyceride	mg per dL	80 (53 - 117)	58 (40 - 83)	0.0035
HDL	High Density Lipoprotein	mg per dL	57 (49 - 69)	64 (58 - 72)	0.0187
Na	Sodium	mM	139 (138 - 141)	140 (138 - 141)	n.s.
K	Potassium	mM	4.0 (3.8 - 4.2)	4.0 (4.0 - 4.3)	n.s.
Cl	Chloride	mM	103 (102 - 104)	104 (102 - 105)	n.s.
ALP	Alkaline Phosphatase	IU	158 (136 - 189)	163 (130 - 189)	n.s.
AST	Aspartate Transaminase	IU	17 (14 - 21)	16 (14 - 19)	n.s.
ALT	Alanine Transaminase	IU	13 (10 - 19)	13 (10 - 15)	n.s.
LD	Lactate Dehydrogenase	IU	153 (134 - 179)	160 (138 - 179)	n.s.
G-GTP	Gamma-Glutamyl Transferase	IU	17 (12 - 22)	16 (12 - 20)	n.s.
CK	Creatine Kinase	IU	67 (53 - 79)	85 (69 - 120)	0.0013
T-BIL	Total Bilirubin	mg per dL	0.6 (0.4 - 0.8)	0.7 (0.5 - 0.9)	0.0310
S.AMY	Serum Amylase	IU	73 (56 - 85)	79 (66 - 101)	n.s.
GLU	Blood Glucose	mg per dL	87 (82 - 91)	85 (82 - 90)	n.s.
ALB	Albumin	g per dL	4.6 (4.4 - 4.8)	4.5 (4.5 - 4.7)	n.s.
HbA1c	Hemoglobin A1c	%	5.3 (5.1 - 5.4)	5.3 (5.2 - 5.5)	n.s.
TSH	Thyroid Stimulating Hormone	µU per mL	2.6 (1.7 - 3.5)	2.0 (1.3 - 2.8)	0.0125
F-T4	Free Thyroxine	ng per dL	1.2 (1.1 - 1.4)	1.2 (1.1 - 1.3)	0.0465
CA	Citric Acid	mg per dL	2.2 (1.8 - 2.5)	2.3 (2.0 - 2.5)	n.s.

Table S2. Metabolite profiling of ME/CFS patients and HC. Result of ^{1}H-NMR metabolomics. Names of metabolites, their corresponding peaks (ppm), and intensity (median and interquartile range in parentheses) are shown for myalgic encephalomyelitis/chronic fatigue syndrome (ME/CFS) patients (n=48) and healthy controls (HC) (n=51). *P* values were determined by two-tailed Mann-Whitney U-test. *P*-values were corrected for multiple testing using Benjamini-Hochberg false discovery rate (FDR) method after FDR adjustment at 0.20. *P* values are as indicated, or not significant (n.s.). ROI refers to region of interest.

Metabolite	ppm	ME/CFS	HC	*P* value
Acetate	1.92	0.150 (0.130 - 0.178)	0.161 (0.137 - 0.197)	n.s.
Alanine (1)	1.48	0.182 (0.125 - 0.217)	0.172 (0.129 - 0.209)	n.s.
Alanine (2)	3.78	0.185 (0.165 - 0.207)	0.176 (0.160 - 0.189)	n.s.
Butyric acid	0.90	0.161 (0.125 - 0.199)	0.150 (0.125 - 0.205)	n.s.
Ethanol	1.18	0.117 (0.075 - 0.189)	0.091 (0.052 - 0.161)	n.s.
Formic acid	8.45	0.176 (0.160 - 0.211)	0.185 (0.151 - 0.213)	n.s.
Glucose (1)	3.90	0.155 (0.050 - 0.240)	0.145 (0.072 - 0.220)	n.s.
Glucose (2)	3.71	0.169 (0.097 - 0.217)	0.154 (0.086 - 0.218)	n.s.
Glucose (3)	3.83	0.177 (0.120 - 0.221)	0.173 (0.128 - 0.207)	n.s.
Glucose (4)	3.40	0.169 (0.069 - 0.235)	0.152 (0.062 - 0.211)	n.s.
Glucose (5)	3.46	0.181 (0.072 - 0.233)	0.157 (0.081 - 0.217)	n.s.
Glucose (6)	3.49	0.175 (0.055 - 0.240)	0.145 (0.063 - 0.209)	n.s.
Glucose (7)	3.53	0.154 (0.051 - 0.228)	0.158 (0.073 - 0.208)	n.s.
Glucose (8)	3.73	0.167 (0.053 - 0.239)	0.151 (0.069 - 0.217)	n.s.
Glycine	3.56	0.165 (0.132 - 0.198)	0.162 (0.143 - 0.202)	n.s.
Hydroxybutyric acid	1.20	0.067 (0.032 - 0.153)	0.073 (0.042 - 0.126)	n.s.
Isoleucine (1)	1.01	0.166 (0.126 - 0.198)	0.162 (0.146 - 0.190)	n.s.
Isoleucine (2)	0.94	0.170 (0.125 - 0.200)	0.159 (0.137 - 0.191)	n.s.
Lactic acid	4.11	0.156 (0.110 - 0.207)	0.183 (0.127 - 0.214)	n.s.
Lactic acid / Threonine	1.33	0.173 (0.114 - 0.216)	0.181 (0.133 - 0.228)	n.s.
Leucine (1)	0.97	0.168 (0.127 - 0.206)	0.174 (0.160 - 0.203)	n.s.
Leucine (2)	0.95	0.173 (0.129 - 0.203)	0.179 (0.158 - 0.207)	n.s.
Methanol	3.35	0.111 (0.081 - 0.178)	0.122 (0.067 - 0.193)	n.s.
Succinic acid	2.37	0.141 (0.071 - 0.239)	0.146 (0.109 - 0.222)	n.s.
Valine (1)	1.04	0.178 (0.140 - 0.211)	0.177 (0.156 - 0.201)	n.s.
Valine (2)	0.99	0.176 (0.142 - 0.207)	0.174 (0.148 - 0.198)	n.s.
ROI.1	0.88	0.179 (0.133 - 0.261)	0.177 (0.123 - 0.223)	n.s.
ROI.2	1.25	0.164 (0.123 - 0.248)	0.184 (0.095 - 0.287)	n.s.
ROI.3	2.04	0.160 (0.129 - 0.191)	0.188 (0.160 - 0.218)	n.s.
ROI.4	3.04	0.156 (0.127 - 0.198)	0.172 (0.136 - 0.221)	n.s.
ROI.5	1.41	0.179 (0.108 - 0.217)	0.183 (0.127 - 0.215)	n.s.

Table S3. Lipoprotein profiling of ME/CFS patients and HC. ^{1}H-NMR-based assessment of lipoproteins. Lipoprotein class, their corresponding peak (ppm), and intensity (median and interquartile range in parentheses) for myalgic encephalomyelitis/chronic fatigue syndrome (ME/CFS) patients (n=48) and healthy controls (HC) (n=51). *P* values were determined by two-tailed Mann-Whitney U-test. *P*-values were corrected for multiple testing using Benjamini-Hochberg false discovery rate (FDR) method after FDR adjustment at 0.20.

Component	ppm	ME/CFS	HC	*P* value
HDL (CP19)	0.837	0.086 (0.070 - 0.095)	0.097 (0.085 - 0.103)	0.0009
HDL (CP22)	0.845	0.085 (0.071 - 0.097)	0.097 (0.084 - 0.105)	0.0012
HDL (CP20)	0.855	0.086 (0.075 - 0.098)	0.095 (0.082 - 0.101)	0.0103
LDL (CP5)	0.87	0.094 (0.089 - 0.100)	0.084 (0.079 - 0.089)	<0.0001
IDL (CP3)	0.878	0.095 (0.081 - 0.110)	0.079 (0.071 - 0.089)	<0.0001
VLDL (CP1)	0.884	0.096 (0.075 - 0.113)	0.074 (0.068 - 0.087)	0.0004
VLDL (CP11)	0.89	0.094 (0.073 - 0.111)	0.074 (0.067 - 0.087)	0.0013
VLDL (CP18)	0.898	0.092 (0.075 - 0.103)	0.079 (0.072 - 0.091)	0.0112

Table S4. List of antibody sets used for FACS analysis in Fig. S2.

Pattern 1

Fluor	Antibody name	Company	Product name	Catalog number	Amount used
FITC	FITC human CD8	BD	HU CD8 FITC MAB 100T	555360	20μL
PE	PE human CD127	BD	HU CD127 PE MAB 0.1MG	557938	5μL
PE-Cy7	PE-Cy7 human CD4	BD	HU CD4 PE-CY7 MAB 100T	348789	5μL
APC	APC human CD25	BD	HU CD25 APC MAB 100T	555434	20μL
V450	V450 human CD3	BD	HU CD3 HRZN V450 MAB 120T	560365	5μL

Pattern 2

Fluor	Antibody name	Company	Product name	Catalog number	Amount used
FITC	FITC human CD19	BD	HU CD19 FITC MAB 100T	555412	20μL
PE	PE human CD14	BD	HU CD14 PE MAB 100T	555398	20μL
PE-Cy7	PE-Cy7 human CD16	BD	HU CD16 PE-CY7 MAB 100T	557744	5μL
APC	APC human CD56	BD	HU CD56 APC MAB 100T	555518	20μL
V450	V450 human CD3	BD	HU CD3 HRZN V450 MAB 120T	560365	5μL

Pattern 3

Fluor	Antibody name	Company	Product name	Catalog number	Amount used
FITC	FITC human CD8	BD	HU CD8 FITC MAB 100T	555360	20μL
PE	PE human CD3	BD	HU CD3 PE MAB 100T	555340	20μL
PE-Cy7	PE-Cy7 human CD4	BD	HU CD4 PE-CY7 MAB 100T	348789	5μL
APC	APC human CD45RO	BD	HU CD45RO APC MAB 100T	559865	20μL
V450	V450 human CD45RA	BD	HU CD45RA HRZN V450 MAB 120T	560362	5μL

Pattern 4

Fluor	Antibody name	Company	Product name	Catalog number	Amount used
FITC	FITC human CD3	BD	HU CD3 FITC MAB 100T	555332	20μL
FITC	FITC human CD14	BD	HU CD14 FITC MAB 100T	555397	20μL
FITC	FITC human CD16	BD	HU CD16 FITC MAB 100T	555406	20μL
FITC	FITC human CD19	BD	HU CD19 FITC MAB 100T	555412	20μL
FITC	FITC human CD20	BD	HU CD20 FITC MAB 100T	555622	20μL
FITC	FITC human CD56	BD	HU CD56 ALEXA488 MAB 100T	557699	5μL
PE	PE human CD123	BD	HU CD123 PE MAB 0.2MG	554529	5μL
APC	APC human CD11c	BD	HU CD11C APC MAB 100T	559877	20μL
V450	V450 human HLA-DR	BD	HU HLA-DR HRZN V450 MAB 50T	561359	5μL

Pattern 5

Fluor	Antibody name	Company	Product name	Catalog number	Amount used
FITC	Anti-TCR δTCS1, Human, Mouse-Mono(TS-1), FITC	Thermo	Anti-TCR δTCS1, Human, Mouse-Mono(TS-1), FITC	TCR2055	5μL
PE	HU VD2 TCR PE MAB 0.1MG	BD	HU VD2 TCR PE MAB 0.1MG	555739	5μL

Pattern 6

Fluor	Antibody name	Company	Product name	Catalog number	Amount used
FITC	FITC human Vbata11	Beckman	TCR Vβ11 TCRBV11S1-FITC	IM1586	20μL
PE	PE human Vaipha24	Beckman	TCR Vα24-PE	IM2283	20μL
